# Mavacamten in hypertrophic obstructive cardiomyopathy with hepato-renal comorbidities: A case report

**DOI:** 10.1097/MD.0000000000046935

**Published:** 2026-01-02

**Authors:** Liping Ma, Fengqin Li, Sophia Dong, Rongrong Li

**Affiliations:** a Department of Cardiovascular Medicine, Shulan (Hangzhou) Hospital, Shulan International Medical College, Zhejiang Shuren University, Hangzhou, P. R. China; b Department of Ultrasound Medicine, Shulan (Hangzhou) Hospital, Shulan International Medical College, Zhejiang Shuren University, Hangzhou, P. R. China; c Department of Biology, Texas A&M University College of Arts and Science, College Station, TX.

**Keywords:** chronic kidney disease, cirrhosis, hypertrophic cardiomyopathy, individualize therapy, mavacamten

## Abstract

**Rationale:**

Mavacamten is an innovative drug for hypertrophic obstructive cardiomyopathy (HOCM). It improves cardiac function by reducing left ventricular outflow tract obstruction. However, safety data in patients with liver and kidney impairment are limited.

**Patient concerns:**

A 68-year-old man was admitted with a history of HOCM, heart failure, gout, and chronic kidney and liver impairment. He was admitted because of worsening heart failure symptoms and an episode of upper gastrointestinal bleeding. The combined burden of these illnesses significantly impaired his ability to perform daily activities independently, leading to hospitalization for optimized management of his advanced cardiac disease.

**Diagnoses:**

Hypertrophic obstructive cardiomyopathy (apical aneurysm, post-cardiac resynchronization therapy), New York Heart Association class III. Hepatic schistosomiasis with portal hypertension, splenomegaly, upper GI bleeding, moderate anemia. Chronic gout with tophaceous deposits. Chronic kidney disease stage IIIb.

**Interventions:**

Initiated mavacamten 2.5 mg once daily for severe left ventricular outflow tract obstruction refractory to maximally tolerated beta-blockers.

**Outcomes:**

The patient showed significant improvement after treatment. The New York Heart Association class decreased from III to I-II, and the LVOTG dropped from 105 to 16 mm Hg. Meanwhile, liver and kidney functions remained stable during the course of treatment.

**Lessons:**

This case highlights the complexity of managing HOCM with multiorgan comorbidities, emphasizing the role of novel therapies (e.g., mavacamten) in refractory outflow obstruction and the need for tailored multidisciplinary approaches.

## 1. Introduction

Hypertrophic obstructive cardiomyopathy (HOCM), a common genetic cardiac disorder, is characterized by mutations in myocardial sarcomere protein genes that disrupt cardiomyocyte alignment and cause asymmetric ventricular wall thickening, ultimately leading to left ventricular outflow tract obstruction (LVOTO). This pathophysiological process manifests clinically through exertional dyspnea, chest pain, and syncope, with severe cases progressing to end-stage heart failure or sudden cardiac death.^[1]^ Diagnosis primarily relies on echocardiography, which reveals hallmark features including increased interventricular septal thickness (≥15 mm in adults) and elevated left ventricular outflow tract pressure gradient (LVOTG) (≥30 mm Hg at rest or ≥ 50 mm Hg with provocation).^[2]^ Additional diagnostic criteria encompass systolic anterior motion of the mitral valve and dynamic LVOTO exacerbated by reduced ventricular preload or increased contractility. Currently, therapeutic strategies have evolved into 3 main categories: traditional pharmacotherapy (beta-blockers and calcium channel blockers remain first-line treatments); interventional procedures (alcohol septal ablation and percutaneous transluminal septal myocardial ablation); and surgical interventions (septal myectomy, the gold standard for drug-refractory cases).

Mavacamten is a selective, allosteric, and reversible small-molecule cardiac myosin inhibitor. It is the first disease-specific treatment for HOCM that targets the key pathophysiological mechanism of the disease. Preclinical studies show that it reduces myosin heads in the “on actin” state, decreasing cross-bridge formation, and shifts myosin to an energy-sparing “off actin” state.^[1]^ Thereby attenuating hypercontractility, alleviating LVOTO, and improving clinical symptoms and quality of life.^[3]^ Structural studies indicate it normalizes disrupted sarcomere protein interactions, restoring function. Phase III clinical trials have demonstrated significant improvements in cardiac functional parameters and exercise capacity, alongside a favorable safety profile.^[4]^ It has been approved by the U.S. FDA (2022) and China NMPA (2024) for use in adults with HOCM classified as New York Heart Association functional class II-III. Mavacamten is initiated at 2.5 mg once daily. Echocardiographic is monitored every 4 weeks. If at week 12, left ventricular ejection fraction (LVEF) remains ≥ 55% and Valsalva-provoked LVOTG is ≥ 30 mm Hg, the dose is increased to 5 mg once daily, with a maximum maintenance dose of 15 mg once daily. However, due to the limited duration of postmarketing surveillance, real-world evidence on therapeutic efficacy in patients with multisystem comorbidities and potential drug-drug interactions remains limited. Prospective observational studies are especially needed in specific populations, including those with hepatic or renal impairment and individuals receiving concomitant cytochrome P450 (CYP450) substrate medications, to comprehensively evaluate long-term clinical outcomes and pharmacovigilance data.

## 2. Case presentation

A 68-year-old Chinese male presented with exertional chest tightness and dyspnea for over a decade, without chest pain, syncope, or presyncope. He was previously diagnosed with HOCM and atrial fibrillation at a local hospital and initiated on metoprolol succinate (2 tablets once daily) sustained-release therapy. In 2018, he underwent cardiac resynchronization therapy implantation for heart failure. Nine days prior to admission, he experienced hematemesis (500–600 mL) with melena, dizziness, and fatigue following non-steroidal anti-inflammatory drug use, prompting transfusion and hemostatic therapy at a local hospital. During treatment, he developed recurrent chest tightness, dyspnea, and diaphoresis, partially relieved by diuretics before transfer to our institution.

**Figure 1.**
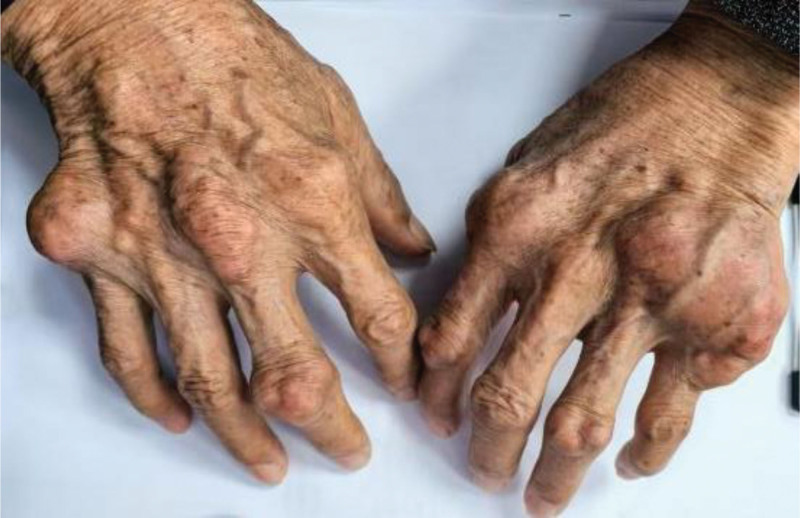
Giant tophi of the hands.

Past medical history included: chronic gout with persistent tophi in both knees, elbows, and digits (Fig. 1), managed intermittently with analgesics; hepatic schistosomiasis with portal hypertension, splenomegaly, and thrombocytopenia (no prior systematic treatment); cholecystectomy; no family history of sudden cardiac death; Chronic alcohol use (30 years, 250 g/d ethanol equivalent) with abstinence for 2 years.

Admission examination: Vital signs: BP 93/62 mm Hg, HR 69 bpm, SpO₂ 98% (room air). Physical findings: Pallor, mild jugular venous distension, bilateral basal lung crackles, grade 5/6 systolic ejection murmur at left sternal border, and multiple gouty tophi with tenderness.

Laboratory results: Renal function: BUN 19.6 mmol/L, Cr 184 µmol/L, eGFR 31.75 mL/min/1.73 m^²^, UA 857 µmol/L. Hematology: Hb 69 g/L, RBC 2.23 × 10^¹²^/L, PLT 62 × 10^⁹^/L. Cardiac biomarkers: BNP > 5000 pg/mL. Liver function: Alb 30.4 g/L, GGT 103 U/L, T-Bil 11 µmol/L (Table 1).

**Table 1:** Patient admission blood indices and echocardiography results.

Form	Value
BUN	19.6 mmol/L
Cr	184 µmol/L
eGFR	31.75 mL/min/1.73m^2^
UA	857 µmol/L
Hb	69 g/L
RBC	2.23 × 10^¹²^/L
PLT	62 × 10^⁹^/L
BNP	>5000 pg/mL
Alb	30.4 g/L
GGT	103 U/L
T-Bil	11 µmol/L
Left atrial diameter	49 mm
IVSd	26 mm
LVOTG	105 mm Hg
LVEF	75%

Alb = albumin, BNP = brain natriuretic peptide, BUN = blood urea nitrogen, Cr = creatinine, eGFR = estimated glomerular filtration rate, GGT = gamma-glutamyl transferase, Hb = hemoglobin, IVSd = interventricular septal thickness at end-diastole, LVEF = left ventricular ejection fraction, LVOTG = left ventricular outflow tract gradient, PLT = platelet, RBC = red blood cell, T-Bil = total bilirubin, UA = uric acid.

**Figure 2.**
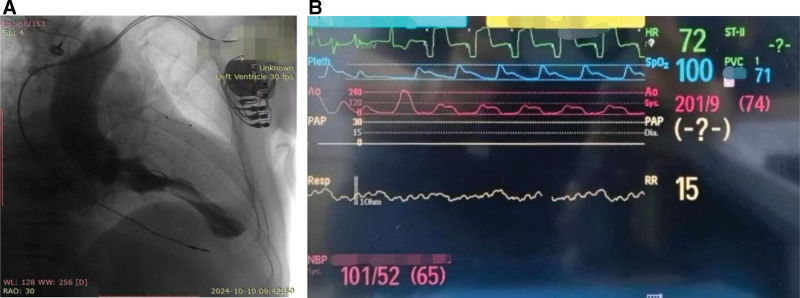
(A) Left ventriculography demonstrated midventricular obstruction with concomitant apical ventricular aneurysm. (B) Continuous hemodynamic monitoring revealed a LVOTG of 100 mm Hg at rest. LVOTG = left ventricular outflow tract pressure gradient.

Imaging: Echocardiography: Left atrial enlargement (49 mm), asymmetric septal hypertrophy (IVSd 26 mm), LVEF 75%, resting LVOTG 105 mm Hg, apical ventricular aneurysm (Table 1). Coronary angiography: Mid-LAD 30% stenosis, left ventricular mid-cavity obstruction, apical aneurysm (Fig. 2). Abdominal ultrasound: Cirrhosis (liver stiffness 35 kPa), splenomegaly. Genetic testing: Heterozygous variant of uncertain significance in MYPN (associated with familial HCM type 22).

Management: Initiated mavacamten 2.5 mg once daily for severe LVOTO refractory to maximally tolerated beta-blockers. Adjusted comorbidities therapy: Diuretics: Furosemide, spironolactone, and tolvaptan 7.5 mg once daily. Anemia: Roxadustat 50 mg once daily, polysaccharide iron complex 0.15 g once daily. Gout: Febuxostat, etoricoxib (as needed). GI prophylaxis: Rabeprazole.

### 2.1. Therapeutic outcomes

Day 13 post-mavacamten initiation: Telephone follow-up revealed marked alleviation of fatigue, with blood pressure improving from 90+/60 + to 129/72 mm Hg.

Serial monitoring of renal/hepatic function, natriuretic peptides, and echocardiography were initiated at 4-week intervals (Table 2).

**Tabel 2:** changes in key parameters from baseline to week 28 of mavacamten treatment.

Week	BP (mm Hg)	T-Bil (µmol/L)	Cr (µmol/L)	BNP (pg/mL)	IVSd (mm)	LVEF (%)	LVOTG (R) (mm Hg)	LVOTG (V) (mm Hg)
W0	93/62	11.0	184	>5000	26	75	105	–
W4	110/72	22.0	125	414	26	74	30	–
W8	116/76	30.7	130	209	25	75	35	71
W12	117/70	29.7	120	151	22	69	21	–
W16	109/65	30.1	127	207	25	72	23	–
W20	118/73	35.1	127	173	22	75	21	50
W24	125/70	34.6	139	129	20	71	13	–
W28	111/73	31.6	193	185	18	75	16	47

BNP = B-Type natriuretic peptide, BP = blood pressure, Cr = creatine, IVSd = interventricular septal thickness in diastole, LVEF = left ventricular ejection fraction, LVOTG(R) = resting Left ventricular outflow tract pressure gradient, LVOTG(V) = valsalva-provoked Left ventricular outflow tract pressure gradient, T-Bil = total bilirubin.

**Figure 3.**
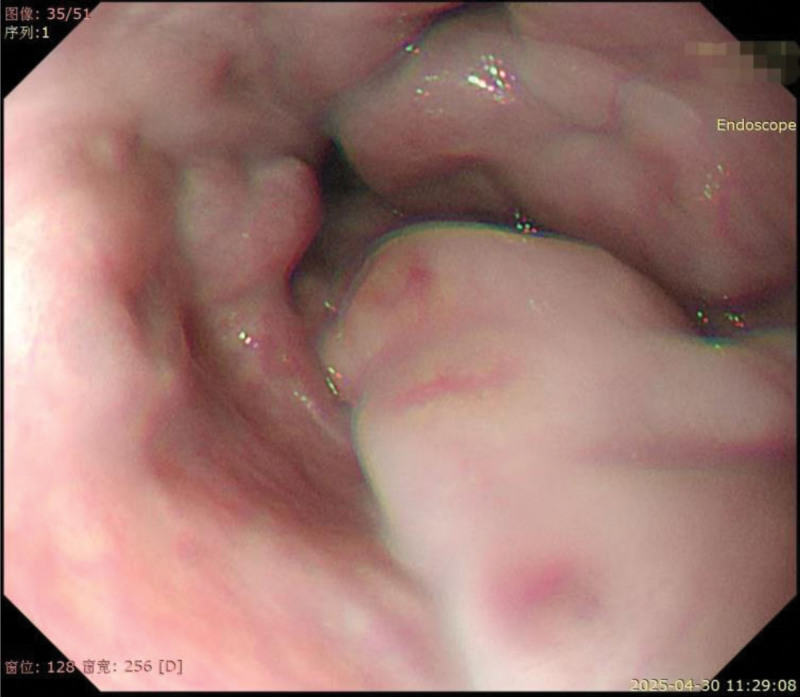
The gastroscopy demonstrated Moderate-to-severe esophageal variceses.

On Day 169, the patient experienced recurrent gastrointestinal bleeding, manifested as hematochezia with fecal occult blood 3+. Laboratory tests showed Hb 114 g/L and PLT 35 × 10^⁹^/L. An emergent esophagogastroduodenoscopy revealed moderate-to-severe esophageal varices (Fig. 3) and portal hypertensive gastropathy. Partial endoscopic variceal ligation was performed on the patient without cardiac decompensation during the periprocedural period.

At the 28-week follow-up, blood indices and echocardiography results were obtained and are summarized in Table 3.

**Table 3:** Patient blood indices and echocardiography results at week 28.

Form	Value
BUN	12.5 mmol/L
Cr	193 µmol/L
eGFR	31.75 mL/min/1.73m²
UA	240 µmol/L
Hb	102 g/L
RBC	3.58 × 10¹²/L
PLT	57 × 10⁹/L
BNP	185 pg/mL
Alb	37.4g/L
GGT	68 U/L
T-Bil	31.6 μmol/l
Left atrial diameter	50 mm
IVSd	18 mm
LVOTG	16 mm Hg
LVEF	75%

Alb = albumin, BNP = brain natriuretic peptide, BUN = blood urea nitrogen, Cr = creatinine, eGFR = estimated Glomerular Filtration Rate, GGT = gamma-glutamyl transferase, Hb = hemoglobin, IVSd = interventricular septal thickness at end-diastole, LVEF = left ventricular ejection fraction, LVOTG = left ventricular outflow tract gradient, PLT = platelet, RBC = red blood cell, T-Bil = total bilirubin, UA = uric acid.

## 3. Discussion

This article comprehensively elaborates on the therapeutic process and remarkable efficacy of mavacamten in a patient with HOCM complicated by liver cirrhosis with portal hypertension and chronic kidney disease. As a breakthrough drug in precision-targeted therapy for HOCM, mavacamten is a first-in-class cardiac myosin inhibitor that reversibly suppresses the myosin-actin cross-bridge cycle by stabilizing the SRX of β-cardiac myosin, thereby reducing myocardial hypercontractility and improving LVOTO and diastolic dysfunction. The clinical significance of this case lies in providing practical evidence for novel pharmacotherapy in special populations with complex comorbidities. For patients with coexisting cirrhosis related portal hypertension and chronic renal insufficiency, pharmacokinetic alterations (e.g., impaired hepatic metabolism and renal excretion) and diminished organ functional reserve substantially increase therapeutic complexity. Successful management of this case underscores the critical role of the multidisciplinary team model, involving collaborative assessments by experts from cardiology, hepatology, nephrology, and clinical pharmacy. Patient-centered therapeutic individualization should be systematically implemented during clinical deployment of novel therapeutic agents, necessitating the establishment of tailored treatment regimens that integrate pharmacogenomic profiles, comorbidity burdens, and organ functional reserves.

In this case, post-treatment resting LVOTG decreased from 105 mm Hg to 13 mm Hg, with interventricular septal thickness (IVSd) regressing from 26 mm to 18 mm. Concurrently, New York Heart Association functional class improved from III to I-II, accompanied by a > 95% reduction in BNP (>5000 → 129 pg/mL) (Table 2), consistent with outcomes from landmark trials (EXPLORER-HCM, VALOR-HCM).^[5,6]^ Notably, BNP reduction exceeding 30% is strongly prognostic of favorable outcomes. Mavacamten is used as an adjunctive therapy for HOCM; it improves cardiac function, increases exercise tolerance, and reduces the need for surgical intervention. However, the absence of full-dose titration (limited by cost and polypharmacy risks) may have constrained therapeutic optimization, as suggested by the dose-dependent efficacy observed in the PIONEER-HCM trial.^[7]^

This case provides a comprehensive evaluation of dose adjustment strategies for patients with hepatic and renal dysfunction. Mavacamten, primarily metabolized by CYP2C19 and CYP3A4,^[8]^ poses challenges in patients with cirrhosis due to enzyme downregulation, which can significantly increase drug exposure. Although liver function indicators remained stable, close surveillance was necessary because of transient bilirubin elevation (Week 28: T-Bil 31.6 µmol/L). This elevation was mainly attributed to hemodynamic changes following bleeding rather than direct hepatic toxicity.

Regarding renal function, based on the patient's baseline chronic kidney disease stage, a standard dosing regimen was appropriate, as the Prescribing Information stipulates no adjustments for eGFR levels above 30 mL/min/1.73 m^2^. Initial decreases in creatinine levels were noted, which were likely linked to enhanced cardiac output and renal blood flow. However, subsequent fluctuations during bleeding episodes highlighted the multifaceted nature of renal susceptibility, exacerbated by diuretic therapy, hyperuricemia, and circulatory instability.

The complexities of comorbid conditions, such as portal hypertension and gastrointestinal bleeding, were effectively managed through endoscopic interventions and cautious maintenance of β-blocker therapy^[9]^ – entailing careful dose adjustment and monitoring – to lower portal pressure. This management strategy necessitated vigilant hemodynamic monitoring due to potential interactions with mavacamten. The normalization of blood pressure after treatment (from 93/62 to 129/72 mm Hg) indicated a favorable response to the alleviation of LVOTO.

Febuxostat has emerged as the preferred agent for managing urate levels in gout patients.^[10]^ It demonstrated significant reductions in UA levels (from 857 to 240 µmol/L), while ECG monitoring showed a concurrent shortening of QTc intervals (from 541 to 525 ms). Short-term use of etoricoxib provided analgesic relief, balancing the cardiovascular risks. Continuous oversight of electrolyte levels and arrhythmia detection through cardiac resynchronization therapy device interrogation ensured patient safety. Follow-up at week 28 found no episodes of ventricular tachycardia.

Long-term safety protocols included rigorous cardiac function surveillance, which revealed no reduction in LVEF (sustained at 75%) despite the reversible negative inotropic effects of mavacamten. This maintenance of LVEF may be attributed to the low dosing regimen and individual pharmacodynamic variability. The choice of rabeprazole over omeprazole was strategic, minimizing potential elevations in mavacamten exposure and managing polypharmacy risks through therapeutic drug monitoring and CYP450 pathway analysis. Dynamic assessments of liver function, adhering to Child-Pugh criteria, along with regular monitoring of creatinine, urate, and eGFR every 4 weeks, facilitated real-time optimization of the treatment regimen.

## 4. Conclusion

Mavacamten is safe for HOCM patients with liver and kidney issues. However, complex cases require multidisciplinary collaboration for personalized management and monitoring. Limited post-marketing surveillance necessitates more clinical studies to strengthen real-world evidence, while health-economic evaluations are essential to improve accessibility to this therapy for more HOCM patients.

## Acknowledgments

The authors are grateful to the patient for giving his permission to describe his clinical case.

## Author contributions

**Conceptualization:** Liping Ma.

**Data curation:** Fengqin Li, Rongrong Li.

**Resources:** Liping Ma.

**Validation:** Liping Ma.

**Visualization:** Fengqin Li, Sophia Dong.

**Writing – original draft:** Liping Ma, Sophia Dong.

**Writing – review & editing:** Liping Ma.

## References

[1] Braunwald E Saberi S Abraham TP Elliott PM Olivotto I. Mavacamten: a first-in-class myosin inhibitor for obstructive hypertrophic cardiomyopathy. Eur Heart J. 2023;44:4622–33. 37804245 10.1093/eurheartj/ehad637 PMC10659958

[2] Reyes KRL Bilgili G Rader F. Mavacamten: a first-in-class oral modulator of cardiac myosin for the treatment of symptomatic hypertrophic obstructive cardiomyopathy. Heart Int. 2022;16:91–8. 36741099 10.17925/HI.2022.16.2.91 PMC9872784

[3] Hegde SM Lester SJ Solomon SD. Effect of mavacamten on echocardiographic features in symptomatic patients with obstructive hypertrophic cardiomyopathy. J Am Coll Cardiol. 2021;78:2518–32. 34915982 10.1016/j.jacc.2021.09.1381

[4] Vanhaecke P Bohbot Y Di Lena C. Usefulness of mavacamten in the challenging association of aortic stenosis and obstructive hypertrophic cardiomyopathy. JACC Case Rep. 2024;29:102430. 39157569 10.1016/j.jaccas.2024.102430 PMC11328762

[5] Olivotto I Oreziak A Barriales-Villa R.; EXPLORER-HCM study investigators. Mavacemten for treatment of symptomatic obstructive hypertrophic cardiomyopathy (EXPLORER-HCM): a ransomised, double-blind, placebo-controlled, phase 3 trial. Lancet. 2020;396:759–69. 32871100 10.1016/S0140-6736(20)31792-X

[6] Desai MY Owens A Wolski K. Mavacamten in patients with hypertrophic cardiomyopathy referred for septal reduction: week 56 Results from the VALOR-HCM randomized clinical trial. JAMA Cardiol. 2023;8:968–77. 37639243 10.1001/jamacardio.2023.3342 PMC10463171

[7] Heitner SB Jacoby D Lester SJ. Mavacamten treatment for obstructive hypertrophic cardiomyopathy: a clinical trial. Ann Intern Med. 2019;170:741–8. 31035291 10.7326/M18-3016

[8] Perera V Gretler D Seroogy J. Effects of omeprazole and verapamil on the pharmacokinetics, safety, and tolerability of mavacamten: two drug-drug interaction studies in healthy participants. Clin Pharmacol Drug Dev. 2023;12:1241–51. 37771180 10.1002/cpdd.1332

[9] Yintao H Ai L Xiao Q Zhang X. Research progress in the treatment of portal hypertensive gastropathy. Adv Clin Med. 2023;13:17428–33.

[10] Xiaofeng Z. 2016 Chinese gout diagnosis and treatment guidelines. Zhejiang Med. 2017;39:1823–32.

